# Modelling the associations between academic engagement, study process and grit on academic achievement of physical education and sport university students

**DOI:** 10.1186/s40359-023-01454-2

**Published:** 2023-11-28

**Authors:** Amayra Tannoubi, Frank Quansah, Iteb Magouri, Nasr Chalghaf, Tore Bonsaksen, Medina Srem-Sai, John Elvis Hagan, Ciptro Handrianto, Fairouz Azaiez, Nicola Luigi Bragazzi

**Affiliations:** 1 Higher Institute of Sport and Physical Education of Kef, University of Jendouba, Jendouba, Tunisia; 2 Higher Institute of Sport, and Physical Education of Sfax, University of Sfax, Sfax, Tunisia; 3 Postgraduate School of Public Health, Department of Health Sciences, University of Genoa, Genoa, Italy; 4 Group for the Study of Development and Social Environment, Faculty of Human and Social Science of Sfax, Sfax, Tunisia; 5 Department of Educational Foundations, University of Education, Winneba, Ghana; 6 Department of Education, Higher Institute of Sport, and Physical Education of Gafsa, University of Gafsa, Gafsa, Tunisia; 7 Department of Health and Nursing Science, Faculty of Social and Health Sciences, Inland Norway University of Applied Sciences, Elverum, Norway; 8 Department of Health, Faculty of Health Studies, VID Specialized University, Stavanger, Norway; 9 Department of Health, Physical Education, Recreation and Sports, University of Education, Winneba, Ghana; 10 Department of Health, Physical Education and Recreation, University of Cape Coast, PMB, Cape Coast, Ghana; 11 Neurocognition and Action-Biomechanics-Research Group, Faculty of Psychology and Sports Science, Bielefeld University, Bielefeld, Germany; 12 Faculty of Human Development, Sultan Idris Education University, Tanjong Malim, Malaysia; 13 Department of Neuroscience, University of Genoa, Genoa, Italy; 14 Laboratory for Industrial and Applied Mathematics, York University, Toronto, ON Canada

**Keywords:** Learning outcomes, Performance, Higher education, Student Engagement, Educational psychology

## Abstract

**Objective:**

The present study examined the impact of academic engagement, study processes, and grit on the academic achievement of physical education and sport university students.

**Methods:**

An internet-based survey recruited 459 university students aged 19–25 years (M = 21 ± 1.3) in physical education and sports (PES) to fill out questionnaires on Physical Education-Study Process Questionnaire (PE-SPQ), Physical Education-Grit (PE-Grit), academic engagement (A-USEI), and Grade Point Average (GPA). A path analysis was carried out to understand variable relationships.

**Results:**

Data from each variable exhibited symmetrical and normal distribution, as indicated by the skewness and kurtosis values. The model’s fit indices showed sufficient Comparative Fit Index (CFI = 0.92), Tucker-Lewis Index (TLI = 0.90), Goodness of Fit Index (GFI = 0.99) and Normed Fit Index (NFI = 0.90) and showed acceptable levels. The results indicated a statistically significant positive impact of engagement (*β =* 0.299, *p <* 0.001) and study processes (*β =* 0.397, *p <* 0.001) on academic achievement. However, the effect of grit on achievement was non-significant.

**Conclusions:**

Academic engagement as well as study processes are two important factors predicting academic achievement while grit seems to be not a major predictor. Hence, physical education and sport faculty and university administrators should prioritize student engagement as a determinant of academic outcomes by reforming or redesigning physical education and sport curriculum modules that can facilitate engagement.

## Introduction

Nowadays, academic success or achievement continues to be a challenge for educators and researchers because it is a complex, multi-faceted phenomenon [1] influenced by a variety of institutional, individual, and situational factors [2]. Studies suggest that students’ intrinsic motivation, self-regulated learning strategies, and metacognitive skills are key factors in academic success [3–7]. Additionally, opportunities for convenient learning experiences and effective instructional practices are also key determinants of student success. As such, understanding these factors can help universities design effective interventions to improve and promote student retention and achievement [8, 9]. Moreover, previous studies have indicated that academic achievement is a critical measure of student success [10, 11]. Other studies suggest that the quality of teaching [12, 13], student engagement in their courses, and study habits are key determinants of academic success [14, 15]. Furthermore, students’ access to technology, resources, and support from their peers and instructors can significantly improve their performance in the classroom [16, 17]. In addition, the classic conceptualization of academic achievement as a variable is that of the Grade Point Average (GPA) [18], which is a common indicator of learner academic success, could be indicative of academic achievement and attainment of educational goals at the university level [19].

Academic achievement results from the interaction of a variety of factors, including engagement, study approach to learning, and grit. Engagement, which reflects the level of cognitive, emotional and behavioral investment in academic study, positively affects academic outcomes [20]. According to Kuh and Hu (2001), the concept has also been defined as how students strive to perform educational activities to achieve desired outcomes [21]. In addition, effective learning approaches, such as active learning and goal setting [22], have been shown to increase student performance in various courses and academic fields [23, 24]. These effective approaches have been defined by several authors as the way students manage their study tasks [25–27], they are also the methods used by a student to acquire, retain and learn knowledge to better perform in exams [28]. It is then commonly reported that there are two distinct study approaches, generally known as the “deep approach” and the “surface approach” [28–30], where the former has been linked to favorable learning outcomes and exam results.

Grit, defined as the combination of passion and determination in the pursuit of long-term objectives, has been linked to improved academic achievements. It serves as a crucial indicator of university completion [31, 32]. It is a relatively recent educational term, which is emerging as an increasingly important factor in setting students up for success in school and in life [33]. Yet, prior research brought up the issue about whether grit is effective for performance and achievement, emphasizing the need for a refined trait [34, 35], and stating their positive association with academic performance which requires following a path of sequential mediators [19, 36]. Possibly, such mediators can be study process and academic engagement.

The scientific study of the interaction between engagement, study process, grit, and academic achievement in a university setting could be critical to understanding how these factors can be harnessed to improve student success [37–40]. This interaction could be important because it helps us understand how these different factors operate in concert to influence student success. By investigating how these factors relate to each other, we may identify the most effective ways to support students in their studies. For example, a highly academically engaged student who developed effective study processes may still have difficulty with academic challenges [41, 42], such as difficult academic coursework or a demanding professor [43]. However, if the student has high grit and resilience, they are more apt to persist through those challenges and to succeed academically [44]. Alternatively, a student lacking engagement or efficient study processes could struggle to succeed, despite having a high level of grit [34].

Multiple theories of personality and human motivation can elucidate the correlation between study processes, grit, and academic engagement for academic success. One of these theories is self-determination theory (SDT) [45]. The SDT suggests that the interplay between study processes, grit, and academic engagement for academic success can be best outlined by the satisfaction of the core psychological needs of competence, autonomy, and relatedness [18, 45–48].

In the study context, the academic achievement of university students in physical education (PE) has recently been of increasing concern as many factors negatively impact their performance [49]. These include lack of motivation, inadequate funding, poor teacher quality, time constraints, and lack of relevance to their future careers [50–53]. As a result, students may struggle to acquire the necessary knowledge and skills in PE, resulting in lower overall academic achievement. This issue underscores the need for universities to address these factors and provide students with the resources and support necessary to succeed in PE and related fields. Nevertheless, studying the relationships between engagement, study approaches and grit, and the effect of these different factors on the academic success of university students in physical education and sport (PES), requires adequate and contextually appropriate measurement scales, such as the grit scale in physical education (PE-Grit [54]), the Physical Education Study Process Questionnaire (PE-SPQ [24]) and the University Student Engagement Inventory (A-USEI [55]). The study of the relationships between these key variables could be beneficial for better understanding of the parameters involved in high performance academic achievement. Thus, the present study examined the impact of academic engagement, study processes, and grit on the academic achievement of university students in PES through path analysis. It was hypothesized that (i) academic engagement with its three factors (emotional, cognitive and behavioral), (i) orientation towards a deep or surface, practical or theoretical study process and (iii) grit with its four domains positively influence students’ academic achievement.

## Materials and methods

### Participants and data collection

An internet-based sample of PES students (*n =* 488) was recruited. Participants were enrolled in the Bachelor of PE program at the Institute of Physical Education and Sports of Kef Tunisia. Students who failed to pass all exams were excluded from the study. An ‘a priori’ power analysis was performed using the G*Power software (Version 3.1.9.7, University of Kiel, Kiel, Germany) and the F-test family (Linear multiple Regression: Fixed model, R^2^ deviation from zero) [56]. The sample size in structural equation modelling is an important consideration because it relates to the discrepancies between the estimated and asymptotic parameters, which affect the estimated statistical power [57]. The analysis revealed that a minimum sample size of 119 participants would be adequate to detect differences (effect size f2 = 0.15, α = 0.05) with an actual power of 95%. In this case, several studies have reported that a sample size of 200 participants only had 0.33 power to detect an indirect effect of 0.06 in the model [57, 58], and a sample size of 460 participants would have 0.81 power to detect the targeted effect. Therefore, the sample size in this study (n = 488) was considered adequate for the applied path analysis. The participants were carefully invited to participate in the study through Facebook or e-mail. An electronic version of the questionnaire was distributed online using Google forms® (Google, California, USA).

Participants were between the ages of 19 and 25 years. The gender proportions were practically equal (female participants, *n =* 252, 51.6%; male participants, *n =* 236, 48.3%) and the average age for the sample was 21 years (women, M = 21 ± 1.3 years; men, M = 20.8 ± 1.35 years). Participants were also divided according to their GPA (1 “Pass” [*n =* 138], 2 “moderate” [*n =* 253] to 3 “high” [*n =* 97]). Then, outliers that could potentially bias the study results were eliminated (*n =* 29). In all, data from 459 students were retained.

### Instruments

#### Grade point average (GPA)

The GPA were used to conceptualize the academic achievement of the students. The GPA represents the average of all final course grades in a program, weighted by the unit value of each course. Our study exclusively recorded students with GPAs exceeding 10, as our focus was primarily on those who had successfully passed their courses. Students participating in the study were enrolled in the bachelor’s degree program, which consists of three years of study.

#### Physical education study process questionnaire (PE-SPQ)

The PE-SPQ with 20 items in Arabic language assessed the processes associated with the study in four context-specific tasks, each with 5-items, was used: [Deep Theory Task (DTT) and Surface Theory Task (STT) Deep Practice Task (DPT) and Surface Practice Task (SPT)] [24]. The questionnaire was generated from the initial version of the R-SPQ-2 F [30], and the Arabic-language version that was validated with university students [59]. The McDonald internal consistency indices across all four PE-SPQ components ranged from 0.86 to 0.94. These results indicate that all four components of the scale exhibit excellent internal consistency. In addition, Cronbach’s α values ranged from 0.86 to 0.93 [24]. Each item on the instrument was scored on a 5-point Likert scale ranging from 0 to 4, with 4 being the highest score (Deeper) and 0 being the lowest (more surface). A confirmatory factor analysis (CFA) was first carried out to assess the validity of the instrument with the participants. Results showed Cronbach’s Alpha reliability coefficients ranging from acceptable (0.73) to excellent (0.90). the calculation of the fit indices showed a suitable model (CFI = 0.95, TLI = 0.94).

#### Physical education grit scale (PE-Grit)

The PE-Grit measurement scale consisting of 16 items in Arabic language [54] measures Grit across four specific dimensions, each composed of four items: Physical Interest (PHI), Physical Effort (PHE), Academic Interest (AI) and Academic Effort (AE) [54]. The internal consistency index usingMcDonald Omega reliability estimate for the four dimensions ranged from 0.83 to 0.86. In addition, Cronbach’s α values for the four factors were good ranging from 0.80 to 0.86 and an overall estimate of 0.83 was achieved [54]. A 7-point Likert scale was used to measure each item, ranging from 0 strongly disagree (no gritty) to 6 strongly agree (extremely gritty). In addition, the results from the CFA showed acceptable values : 0.96 and 0.95 for the CFI and TLI, respectively. Thus, ensuring that the Arabic version of the PE-Grit used with our participants is valid measure for assessing grit.

#### Arabic University Student Engagement Inventory (A-USEI)

The A-USEI was used to measure academic engagement. The instrument is a Likert-type self-report scale with responses ranging from (1 = “never”) to (5 = “always”), consisting of 15 items divided into three dimensions of academic engagement: behavioral (BE), cognitive (CE) and emotional (EE) [55]. The inventory has shown good evidence of reliability and factorial validity in previous studies [60–63].

For the Arabic version of the scale, exploratory and confirmatory factor analyses have been used to methodically validate the specific items for each dimension. The reliability coefficient (Cronbach α) indicating internal consistency between scale items are acceptable and ranged from 0.70 to 0.86 for all three dimensions [55]. The CFA results confirmed the validity of the A-USEI with our participants. Thus, calculation of the Cronbach’s α coefficient of reliability showed values ranging from acceptable to good (0.77–0.82), and a good model fit indices (CFI = 0.96; TLI = 0.95).

### Ethical statement

Approval for this study was obtained from the local ethics committees of the “High Institute of Sport and Physical Education of El Kef, University of Jendouba, Jendouba, Tunisia” and “High Institute of Sport and Physical Education of Sfax, University of Sfax, Sfax, Tunisia”. Further, the study procedures adhered to the most recent legal requirements specified in the “Declaration of Helsinki 2013” [64]. An informed consent form was received and signed by each of the participants before completing the questionnaires. There was no requirement for them to join the study and were informed that there was no need to justify any refusal to do so.

### Statistical analysis

Quantitative analyses were performed using SPSS 27.0 statistical software (IBM corps., Armonk, NY, USA) and free JASP 0.17 software incorporating the Lavaan package of R software (JASP Team, 2023; JASP Version 0.17). The initial analysis of the quantitative data was performed to check for anomalies, missing values and any irregularities in the collected data. We performed a univariate analysis to assess skewness and kurtosis, and conducted multivariate normality tests using the Mardia coefficient. Additionally, the descriptive statistics for each variable was computed. Then, the measure of model fit used to observed data in a structural covariance model was expressed by the following indices: the Comparative Fit Index (CFI), Tucker-Lewis Index (TLI) and Bentler-Bonett Normed Fit Index (NFI). These indices assess the fit of the model by comparing it to a null model. According to Hu and Bentler [65], the values of CFI, TLI and NFI vary from 0 to 1, with values above 0.90 generally indicating a good model fit. In addition, the root mean square error of approximation (RMSEA) measures the fit of the model by comparing the covariance matrix of the model to the observed data, adjusting for the number of model parameters. The RMSEA ranges from 0 to 1, with values below 0.05 generally indicating a good fit of the model to the data [65]. The Goodness of fit index (GFI) measures the proportion of variance and covariance in the observed data that are reproduced by the model. The GFI varies from 0 to 1, with values close to 1 indicating a good fit of the model to the observed data [66]. Subsequently, structural validity was checked using factor loadings indicating the correlation between each manifest (observed) variable and the corresponding latent (unobserved) factor in structural covariance modeling. The factor loadings are measures of the influence or contribution of each variable to the latent factor. Their magnitude indicates the strength of the relationship between each variable and the latent factor [67]. Positive values indicate that the manifest variable is positively correlated with the latent factor, while negative values indicate an inverse correlation [67, 68]. Additionally, negative values are associated with lower values for log likelihood ratio (LLR), Akaike’s information criterion (AIC), and the Bayesian information criterion (BIC) [69]. Regression coefficients were calculated to identify the magnitude and direction by which an independent variable and a dependent variable are related in a regression model [70, 71].

## Results

### Descriptive statistics

The descriptive results for each study variable are presented in Table 1.

The variables are Behavioral Engagement (BE), Emotional Engagement (EE), Cognitive Engagement (CE), Physical Interest (PHI), Physical Effort (PHE), Academic Interest (AI), Academic Effort (AE), Surface Practical Task (SPT), Surface Theoretical Task (STT), Deep Theoretical Task (DTT) and Deep Practical Task (DPT).

The distribution of each variable seems to follow a normal distribution based on the kurtosis values ranging from − 1 to 1 and the skewness measures ranging from − 2 to 2. The multivariate Mardia’s coefficient (4.88, z = 3.36, p < 0.01) suggested that the data exhibited satisfactory multivariate normality.

**Table 1:** Descriptive analysis of variables

	Mean	Std. Deviation	Skewness	Kurtosis
BE	2.738	0.579	0.388	-0.155
EE	2.675	0.694	0.335	-0.146
CE	2.600	0.663	0.363	-0.314
PHI	3.087	0.833	-0.060	-0.493
PHE	2.948	0.846	-0.038	-0.790
AI	2.709	0.865	0.212	-0.525
AE	2.769	0.913	0.272	-0.571
SPT	3.013	0.816	-0.104	-0.451
DPT	2.871	0.808	0.249	-0.788
STT	2.586	0.790	0.438	-0.442
DTT	2.569	0.852	0.527	-0.313

[DPT: Deep Practical Task; DTT: Deep Theoretical Task; SPT: Surface Practical Task; STT: Surface Theoretical Task; BE: Behavioral Engagement; EE: Emotional Engagement; CE: Cognitive Engagement; PHI: Physical Interest; PHE: Physical Effort; AI: Academic Interest; AE: Academic Effort]

### Fit indices

Table 2 presents the model fit indices, which assess the model’s adequacy to the observed data. The Comparative Fit Index (CFI = 0.92),  the Tucker-Lewis Index (TLI = 0.90) and the Root Mean Square Error of Approximation (RMSEA) value of 0.08 indicate acceptable model fit. In addition, the Goodness of Fit Index (GFI) and Bentler-Bonett Normed Fit Index (NFI) values are greater than 0.9, indicating a good model fit. The T-size RMSEA is computed for α = 0.05. The T-size equivalents of the conventional RMSEA cut-off values (close < 0.05 < fair < 0.08 < poor) are close < 0.062 < fair < 0.091 < poor for model.

**Table 2:** Model fit indices

Fit indices	
Index	Value
Comparative Fit Index (CFI)	0.92
Tucker-Lewis Index (TLI)	0.90
Goodness of fit index (GFI)	0.99
Bentler-Bonett Normed Fit Index (NFI)	0.90
Root mean square error of approximation (RMSEA)	0.08
Log-likelihood (LLR)	-5503.476
Akaike (AIC)	11.088.95
Bayesian (BIC)	112058.243

### Parameters estimation

Table 3 presents the factor loadings for a three-factor measurement model consisting of Engagement (ENG), Grit (GRIT), and Study Process Questionnaire (SPQ) with indicators specified for each factor. The factor loadings represent the correlation between each indicator and its corresponding latent factor. The 95% confidence intervals for each factor loading are also presented.

**Fig. 1:**
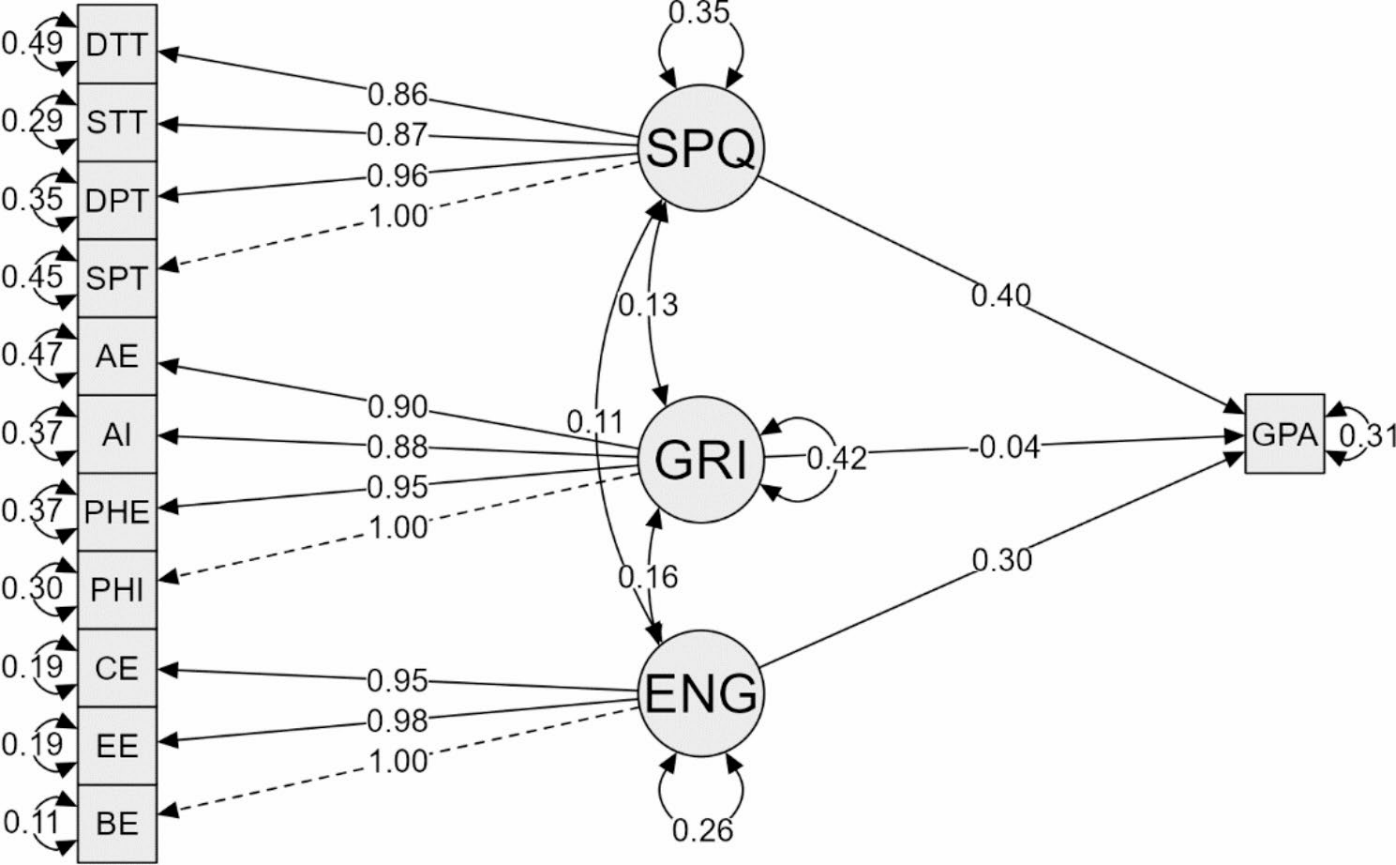
Path model of the relationship between GPA, SPQ, ENG, and GRIT and the different factors of each measure

For example, the factor loadings for the indicator (EE) on the factor (ENG) is 0.982 with a confidence interval of 0.964 to 1. This means that the (EE) indicator is correlated with the (ENG) factor. Similarly, the factor loading for the (PHE) indicator on the (GRIT) factor is 0.954 with a confidence interval of 0.931 to 0.978, suggesting a correlation between these two variables. All factor loadings as well as the relationships between the different variables are presented in a three-factor path model (Fig. 1).

**Table 3:** Factor loadings of the constructed model

						95% Confidence Interval		Standardized		
Latent	Indicator	Estimate	Std. Error	z-value	p	Lower	Upper	All	LV	Endo
ENG	BE	1.000	0.000			1.000	1.000	0.838	0.510	0.838
	EE	0.982	0.009	107.017	< .001	0.964	1.000	0.757	0.501	0.757
	CE	0.952	0.009	103.700	< .001	0.934	0.970	0.741	0.486	0.741
GRIT	PHI	1.000	0.000			1.000	1.000	0.762	0.645	0.762
	PHE	0.954	0.012	79.799	< .001	0.931	0.978	0.712	0.616	0.712
	AI	0.882	0.012	75.799	< .001	0.859	0.904	0.682	0.569	0.682
	AE	0.899	0.013	71.017	< .001	0.875	0.924	0.644	0.580	0.644
SPQ	SPT	1.000	0.000			1.000	1.000	0.661	0.589	0.661
	DPT	0.959	0.013	71.592	< .001	0.933	0.986	0.693	0.565	0.693
	STT	0.869	0.012	71.104	< .001	0.845	0.893	0.692	0.512	0.692
	DTT	0.858	0.014	61.535	< .001	0.831	0.885	0.587	0.506	0.587

[ENG: Engagement; SPQ: Study Process Questionnaire]

### Model’s regression coefficients

Table 4 presents the regression coefficients for the predictors (ENG, GRIT, SPQ) on the dependent variable (GPA), with 95% confidence intervals (Standardized values). The results indicate that there was a significantly positive effect of ENG (Estimate = 0.299, p < 0.001) and of SPQ on GPA (Estimate = 0.397, p < 0.001), whereas the effect of GRIT was not significant (p > 0.05). The confidence intervals suggest that these estimates are accurate with a small margin of error.

**Table 4:** Model’s Regression Coefficients

						95% Confidence Interval	
Predictor	Outcome	Estimate	Std. Error	z-value	p	Lower	Upper
ENG	GPA	0.299	0.070	4.293	< 0.001	0.162	0.435
GRIT	GPA	0.037	0.056	0.657	0.511	0.146	0.073
SPQ	GPA	0.397	0.055	7.227	< 0.001	0.289	0.504

[ENG: Engagement; SPQ: Study Process Questionnaire]

## Discussion

The present study examined the impact of academic engagement, study processes, and Grit on the academic achievement of PES university students through path analysis. Model fit indices to the observed data indicated acceptable model fit. Findings from factor loadings calculation resulted in a three-factor measurement model (ENG, GRIT, SPQ) with acceptable factor loadings. The results showed high correlations between some indicators and their corresponding latent factor, such as the Emotional Engagement indicator (EE) with the Engagement factor (ENG). Other indicators had more moderate correlations with their latent factor, such as the Physical Effort indicator (PHE) with the factor (GRIT). These findings align with previous studies that have determined a positive correlation between intense physical activity and resilience, as well as the perseverance of effort grit [72]. This suggests that grit could be a suitable focus for ensuring the long-term effectiveness of physical activity interventions [73]. Moreover, the findings derived from the estimation of the factor loadings in our regression model indicate that the theoretical subscales (DTT and STT) of the SPQ exhibit a higher level of influence on the latent factor in comparison to the practical subscales (DPT and SPT). This finding suggests that students who adhere to a theoretical orientation tend to demonstrate higher levels of academic achievement compared to those who predominantly depend on practical study approaches [74, 75].

Findings of the present research also showed that ENG and SPQ had significant positive effects on GPA, while the effect of GRIT was not significant. The observed results are partly consistent with those of various studies that have shown academic engagement to be associated with study success [76, 77]. Moreover, the study potentially adds to this knowledge by demonstrating that academic engagement is also associated with student success, operationalized by exam grades. Further, academic engagement has a significant effect on student outcomes as expressed in GPA. These results could be explained by the fact that academic achievement depends not only on the volume of study, but also on how students learn (study process) [78, 79] as well as the feelings and attitudes they associate with their studies and habits (engagement) [14, 15, 80].

The findings provided a three-factor explanatory model. As previously presented, the results showed a significant positive effect of ENG and SPQ on the GPA, while the effect of GRIT was not significant. These findings are not in line with the results of previous research conducted with university students in different fields of education, such as educational sciences [81], polytechnics [82] and biology-environmental sciences [83]. These studies showed that the choice of study processes mainly had a significant mediating effect on participants’ academic success. In a recent structural equation modelling (SEM) study of 351 students from Anglophone countries (US, Canada, UK and Israel), academic Grit was found to be directly associated with academic achievement in university students [58].

In summary, the findings of the current study partially support the suggested hypotheses regarding (i) the significant impact of engagement elements and (ii) study processes on success. However, (iii) the Grit did not have any significant impact on students GPA.

A possible explanation for this is that university students in PES are distinguished from other fields by the duality of practical and theoretical tasks [84, 85], in which the student must excel in both components to ensure academic performance [24]. Thus, according to the explanatory model from our study, the non-significant relationship between grit and GPA, could be due to the difficulties and complexity of the academic process in PES that require students to excel in the different practical and theoretical tasks for academic achievement. Similarly, the diversity of physical education students’ personalities and thus their degree of perseverance in practical and theoretical tasks could explain the non-significant association between grit and academic achievement [33]. This study has some limitations. First, the data were collected from a single academic institution, which limits the generalizability of the results to other academic populations or settings. Second, due to the cross-sectional study design, we are unable to conclude about the direction of the detected associations (i.e., causality). For example, cyclical associations are viable – specifically, study processes and academic engagement may impact on academic performance, but exam grades can also impact on students’ engagement and how they handle the study process later on. In addition, the measurement of academic performance was limited to GPA alone, when other indicators of academic success could be relevant, such as exam pass rates, number of credits earned, or additional degree attainment.

## Conclusion

 Students’ academic engagement and study process orientation (Deep or Surface) are important factors in predicting academic performance, while Grit is not. These results may be useful for university teachers and administrators in physical education and sport to understand the factors that influence students’ academic performance. In general, to facilitate good academic performance among PES students, it appears that emphasis should be placed on the students’ own feelings, attitudes and engagement related to their studies.

## Data Availability

The original contributions presented in the study are included in the article. Further inquiries can be directed to the corresponding author.
